# Amelioration of Glucolipotoxicity-Induced Endoplasmic Reticulum Stress by a “Chemical Chaperone” in Human THP-1 Monocytes

**DOI:** 10.1155/2012/356487

**Published:** 2012-04-10

**Authors:** Raji Lenin, Mariawilliam Sneha Maria, Madhur Agrawal, Jayashree Balasubramanyam, Viswanathan Mohan, Muthuswamy Balasubramanyam

**Affiliations:** ^1^Department of Cell and Molecular Biology, Madras Diabetes Research Foundation and Dr. Mohans' Diabetes Specialities Centre, Chennai 600086, India; ^2^Centre for Biotechnology, Anna University, Chennai 600025, India

## Abstract

Chronic ER stress is emerging as a trigger that imbalances a number of systemic and arterial-wall factors and promote atherosclerosis. Macrophage apoptosis within advanced atherosclerotic lesions is also known to increase the risk of atherothrombotic disease. We hypothesize that glucolipotoxicity might mediate monocyte activation and apoptosis through ER stress. Therefore, the aims of this study are (a) to investigate whether glucolipotoxicity could impose ER stress and apoptosis in THP-1 human monocytes and (b) to investigate whether 4-Phenyl butyric acid (PBA), a chemical chaperone could resist the glucolipotoxicity-induced ER stress and apoptosis. Cells subjected to either glucolipotoxicity or tunicamycin exhibited increased ROS generation, gene and protein (PERK, GRP-78, IRE1**α**, and CHOP) expression of ER stress markers. In addition, these cells showed increased TRPC-6 channel expression and apoptosis as revealed by DNA damage and increased caspase-3 activity. While glucolipotoxicity/tunicamycin increased oxidative stress, ER stress, mRNA expression of TRPC-6, and programmed the THP-1 monocytes towards apoptosis, all these molecular perturbations were resisted by PBA. Since ER stress is one of the underlying causes of monocyte dysfunction in diabetes and atherosclerosis, our study emphasize that chemical chaperones such as PBA could alleviate ER stress and have potential to become novel therapeutics.

## 1. Introduction

Cardiovascular complications due to atherosclerotic disease are a frequent cause of morbidity and mortality in patients with diabetes [1]. A role for immune cells such as monocytes and lymphocytes has been implicated in the pathogenesis of diabetes and its various complications including atherosclerosis. Clinical and experimental evidence indicates that inflammatory processes in the vascular wall are the decisive factor that accounts for the rate of lesion formation and clinical development in patients suffering from atherosclerosis [2]. Monocytes and macrophages, in an attempt to engulf lipid laden cells along the blood vessel, will get exposed to hyperglycemia and hyperlipidemia, a combined pathophysiological situation referred to as “glucolipotoxicity.” The exact mechanisms by which glucolipotoxicity triggers monocyte activation and atherosclerotic processes are not clearly understood. However, evidence indicates that ER stress plays an important role in these mechanisms.

ER stress is known to activate a series of signals that comprise the unfolded protein response (UPR). The UPR includes at least three signaling pathways initiated by the kinases IRE1 and PERK and the transcription factor ATF6 [3] signals that coordinate the cellular response to unfolded proteins, which includes (a) downregulation of protein translation, (b) enhanced expression of ER chaperone proteins that promote protein refolding, and (c) activation of proteases involved in the degradation of misfolded proteins. Conversely, prolonged or severe ER stress can lead to the activation of apoptotic cell death. Recent studies have suggested that chronic ER stress originating from glucolipotoxicity is one of the major culprits that can very well explain the underlying causes of both insulin resistance and *β*-cell dysfunction [4–7]. ER stress has been linked to insulin resistance in diabetes and also expansion of ER was detected in **β**-cells from type 2 diabetic patients [8, 9]. Furthermore, increased expression of ER stress markers has been demonstrated in db/db mouse islets and **β**-cells of type 2 diabetes patients [9, 10]. Recent studies also imply that **β**-cell dysfunction by glucolipotoxicity-induced ER stress is mediated by increased oxidative stress and apoptosis [11]. While the recruitment of monocytes and their retention within atherosclerotic lesions contribute to plaque development [12], macrophage apoptosis within advanced atherosclerotic lesions is also known to increase the risk of atherothrombotic disease [13]. Although glucolipotoxicity is known to trigger ER stress, its effect on various arms of the ER stress machinery in monocytes has not been clearly defined. Therefore, we investigated the effect of glucolipotoxicity on monocytes with reference to ROS generation, transcriptional alterations of various ER stress markers, and apoptotic indicators. We also investigated the effect of 4-phenyl butyric acid (PBA, a chemical chaperone) whether it resists glucolipotoxicity-induced ER stress and apoptosis.

## 2. Materials and Methods

### 2.1. Cell Culture and Treatment

The human monocyte THP-1 cells were obtained from the National Centre for Cell Science (NCCS, Pune, India). THP-1 cells were maintained in endotoxin-free RPMI-1640 containing 5.5 mM glucose, 10% (v/v) heat-inactivated fetal bovine serum, 100 U/mL penicillin, 100 *μ*g/mL streptomycin, 2.5 *μ*g/L amphotericin B, 2 mM L-glutamine, 1 mM sodium pyruvate and 10 mM HEPES, pH 7.0–7.4, under a humidified condition of 5% CO_2_ at 37°C. For all the experiments, cells were subjected to either 5.5 mM glucose (basal) or glucolipotoxicity (25 mM glucose plus 0.5 mM palmitic acid) or tunicamycin (4 *μ*M) treatment for 24 h in the presence and absence of 4-PBA (1 mM), in serum-free medium. All experiments were independently performed at least thrice.

### 2.2. Intracellular Reactive Oxygen Species (ROS) Measurement

Intracellular ROS generation was measured in THP-1 monocytes as described earlier [14] with minor modifications as to the confocal application. Briefly, 1 × 10^3^ cells were seeded in confocal chambered slides (LabTek II) precoated with 0.01% poly-L-lysine. After the treatment conditions, 2′,7′-dichlorodihydrofluorescein diacetate (H2-DCFDA, 10 *μ*M) dye was added to each well and incubated for 30 min at 37°C. Cells were then washed twice with PBS. To capture the change in fluorescence, cells were excited at a wavelength of 485 nm and the fluorescence emission was read at 530 nm using Carl Zeiss-LSM 700 confocal microscope with an objective of 20x. Change in mean fluorescence intensity was represented as arbitrary units (AU).

### 2.3. Real-Time PCR

#### 2.3.1. RNA Isolation

Total RNA from cells was isolated as described previously [15]. The RNA quality and concentration of total RNA were measured using nanodrop. 1 *μ*g of RNA was converted to cDNA using 100 units reverse transcriptase enzyme, 40 *μ*M Oligo-dT18 primer (New England Biolabs), 10xRT buffer, 20 U RNase inhibitor (Amersham Biosciences), and 2.5 mM each of dNTPS and incubated at 42°C for 1 h.

#### 2.3.2. Quantitative Real-Time PCR

Quantitative real-time PCR was performed for specific genes using SYBR green master mix (Finnzymes). PCR amplification was carried out using ABI-7000 (Applied Biosystems) with cycle conditions (initial cycle: 50°C for 2 min, Initial denaturation 95°C for 15 sec, 40 cycles of denaturation 95°C for 15 sec, and annealing/extension of 60°C for 1 min). The expression level of RNA was determined using 2^-DDCt^ and normalized using *β*-actin. The primer sequences of specific genes are listed in Table 1.

### 2.4. Protein Expression

After specific treatments, cells were lysed using RIPA buffer (50 mM Tris-HCl (pH 8.0), 150 mM NaCl, 0.1% SDS, 0.2% sodium azide, 1% Triton X-100, 0.25% sodium deoxycholate, and 1x protease inhibitor). In brief, cells were sonicated and incubated for 1 hr in ice and centrifuged at 16,000 g for 5 minutes at 4°C. The supernatant collected was quantified for protein by Bradford method. 15 *μ*g protein was resolved on a 10% SDS-PAGE and transferred to polyvinylidine fluoride (PVDF) membrane. After 1 hour blocking in 5% bovine serum albumin (BSA) and incubation with the appropriate primary antibodies and HRP-conjugated secondary antibodies, detection was performed using enhanced chemiluminescence kit (GE Healthcare). *β*-actin was used as internal control. Mean densitometry data from independent experiments were normalized to control using Image-J software and represented as the ratio of test protein and *β*-actin.

### 2.5. DNA Damage—Comet Assay

DNA damage was evaluated with the Comet assay as described previously [16]. Briefly, the treated cells were mixed with 200 *μ*L of 0.5% low-melting point agarose and layered over clear microscope slides precoated with 1% normal melting agarose. Lysis, electrophoresis, and neutralization were followed by staining with ethidium bromide. The slides were examined under a fluorescent microscope. They were scored using an image analysis system (Comet Imager 1.2.13) attached to a fluorescent microscope (Carl-Zeiss, Germany) equipped with appropriate filter.

### 2.6. Caspase 3 Activity Assay

Caspase-3 activity was determined by colorimetric assay using the caspase-specific peptide containing amino acid sequence Asp-Glu-Val-Asp (DEVD) that is conjugated to the color reporter molecule p-nitroanilide (pNA) (RandD systems). The cleavage of the peptide by the caspases releases the chromophore pNA, which is quantified spectrophotometrically at 405 nm. Cells harvested after treatment were lysed and 10 *μ*g protein was aliquoted from each sample into a 96 well plate. 0.5 *μ*L DTT was added to all the wells followed by addition of 50 *μ*L of 2x reaction buffer and 3.5 *μ*L of Caspase-3 colorimetric substrate. The plate was incubated at 37°C for 1 hr and read at 405 nm using microplate reader. Caspase-3 activity was expressed as mean ± SEM of optical density.

### 2.7. Statistical Analysis

Statistical Package for Social Sciences (SPSS) Windows, (Version 16.0, Chicago, IL), was used for statistical analysis. Data were expressed as Mean ± SEM. Comparisons between groups were performed using student's *t*-test and a *P*-value < 0.05 was considered statistically significant.

## 3. Results

**Figure 1:**
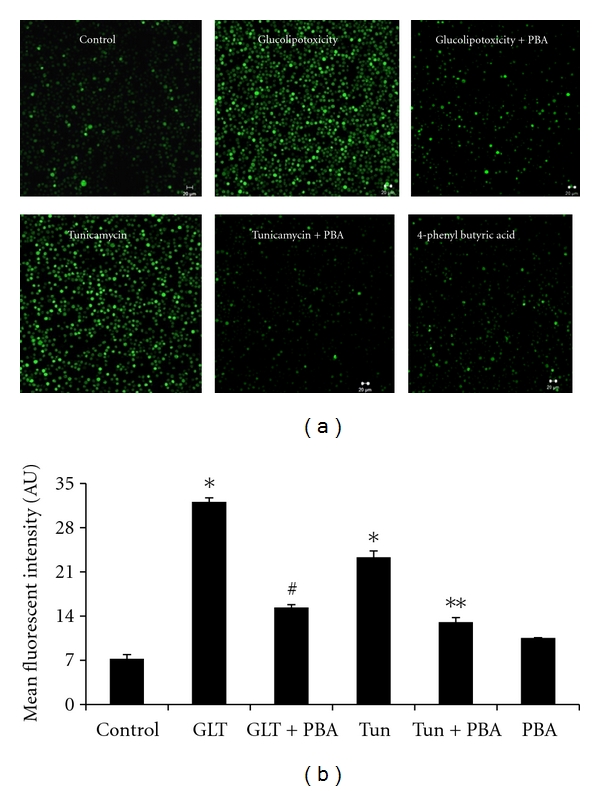
(a) Representative confocal microscopy images of Intracellular ROS generation in cells treated under various conditions. (b) Mean (±SEM) fluorescence intensities of ROS under different experimental maneuvers, namely, control, glucolipotoxicity (GLT), glucolipotoxicity + 4-phenyl butyric acid (GLT + PBA), tunicamycin (Tun), tunicamycin + 4-phenyl butyric acid (Tun + PBA), 4-phenyl butyric acid (PBA). *P < 0.05 compared to control, ^#^ *P* < 0.05 compared to GLT, **P < 0.05 compared to tunicamycin.

**Figure 2:**
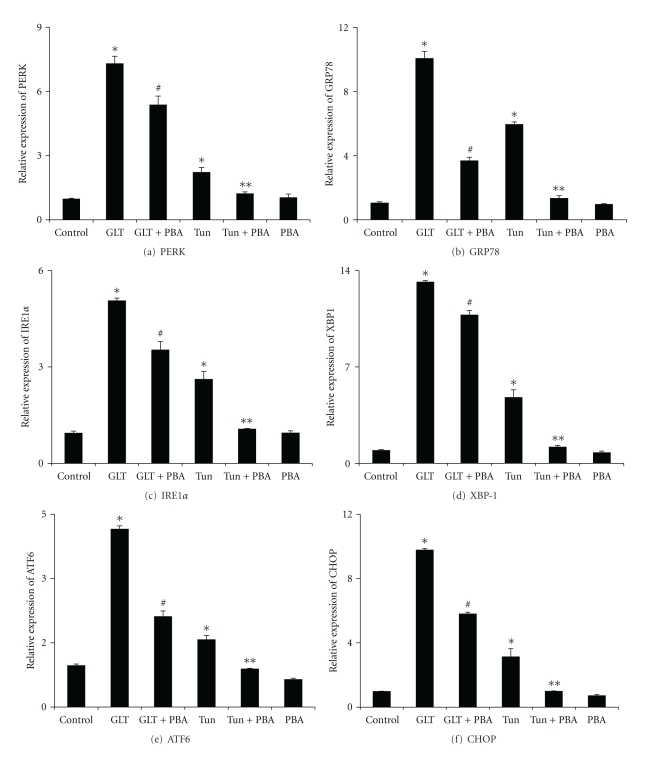
Relative gene expression Mean (±SEM) data of ER stress markers namely, PERK (a), GRP78 (b), IRE1*α* (c), XBP-1 (d), ATF6 (e), CHOP (f). *P < 0.05 compared to control, ^#^ *P* < 0.05 compared to GLT, **P < 0.05 compared to tunicamycin.

**Figure 3:**
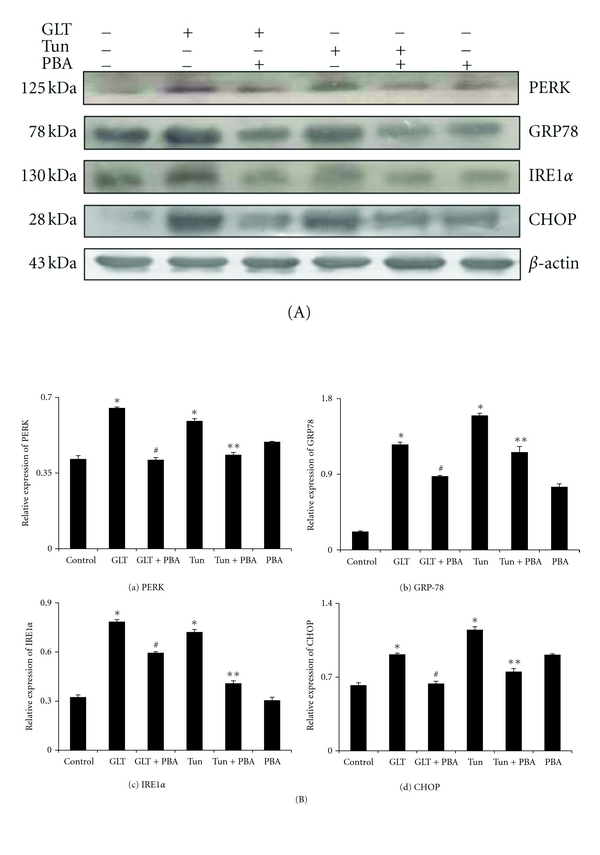
(A) Representative protein expression data on ER stress protein markers, namely, PERK (1), GRP78 (2), IRE1*α* (3), and CHOP (4). (B) Cumulative histogram data Mean (±SEM) of ER stress markers, namely, PERK (a), GRP78 (b), IRE1*α* (c) and CHOP (d). *P < 0.05 compared to control, ^#^ *P* < 0.05 compared to GLT, **P < 0.05 compared to tunicamycin.

We have applied confocal microscopy to measure the fluorescent intensity of DCF which indicates the extent of intracellular ROS generation in monocytes. While representative fluorescent images related to ROS generation were depicted in Figure 1(a), cumulative data on ROS generation were presented in Figure 1(b). Cells subjected to either glucolipotoxicity or tunicamycin exhibited increased ROS generation compared to the untreated cells (Figure 1(b)). Pretreatment of cells with 4-phenyl butyric acid showed significant reduction in ROS generation (*P* < 0.05). A complete mRNA expression pattern of ER stress machinery was determined by real-time PCR and the cumulative data on mRNA pattern of ER stress markers in relation to their ratio with the house keeping *β*-actin was depicted in Figure 2. Compared to untreated cells, cells treated with glucolipotoxicity or tunicamycin showed an increased mRNA expression of PERK, GRP78, IRE1*α*, XBP1, ATF6, and CHOP (Figures 2(a)–2(f)). It is also interesting to note that the extent increase in ER stress markers under glucolipotoxicity was higher than that induced by tunicamycin. PBA treatment significantly reduced all the transcriptional expression of ER stress markers most likely due to its chaperone activity.  Figure 3(A) depicts the representative protein expression of ER stress markers along with *β*-actin. Consistent with the mRNA results, protein expression of ER stress markers, namely, PERK, GRP78, IRE1*α*, and XBP1 were also increased in cells subjected to glucolipotoxicity and tunicamycin treatment (Figure 3(B)). 4-PBA treatment significantly reduced the protein expression of all the ER stress markers.

**Figure 4:**
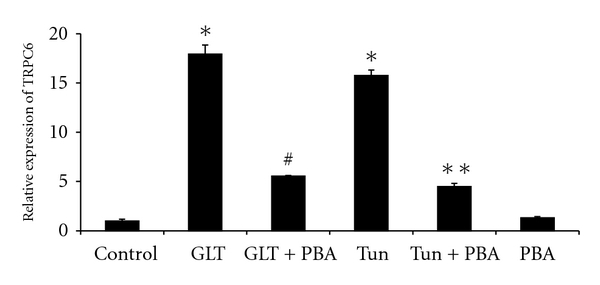
Mean (±SEM) mRNA expression of TRPC-6. *P < 0.05 compared to control, ^#^ *P* < 0.05 compared to GLT, **P < 0.05 compared to tunicamycin.

Since the programming of apoptosis by both the oxidative and ER stress signals depend on the load of intracellular Ca^2+^, we investigated the transcriptional level of TRPC-6, which is an important driving force for increased Ca^2+^ influx. Cells treated with glucolipotoxicity or tunicamycin resulted in several folds of increase in TRPC-6 mRNA levels (Figure 4) and this was significantly normalized by PBA.

**Figure 5:**
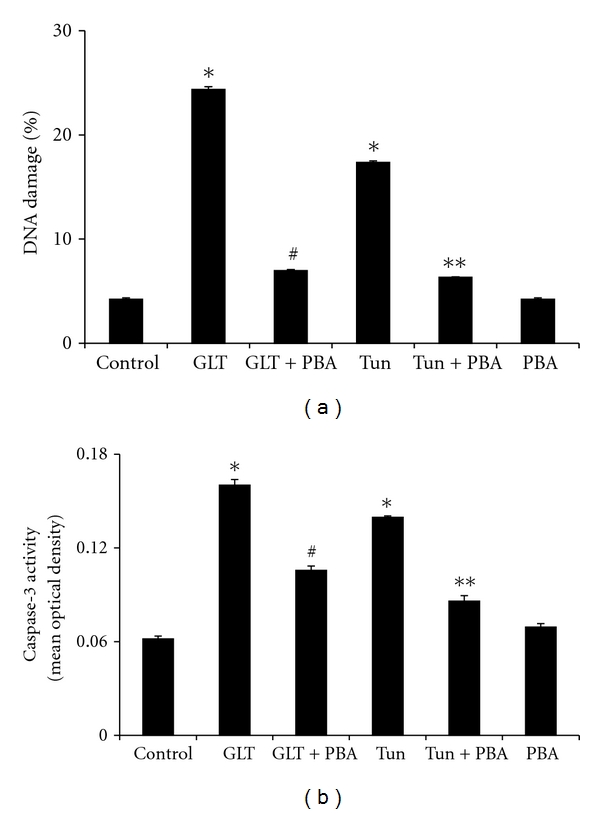
(a) Mean (±SEM) percentage DNA damage under different experimental conditions. *P < 0.05 compared to control, ^#^ *P* < 0.05 compared to GLT, **P < 0.05 compared to tunicamycin. (b) Mean (±SEM) Caspase 3 activity under different experimental conditions. *P < 0.05 compared to control, ^#^ *P* < 0.05 compared to GLT, **P < 0.05 compared to tunicamycin.

Compared to untreated cells, cells treated with glucolipotoxicity or tunicamycin showed increased DNA damage (4.6 and 3 folds, resp.). Interestingly, PBA treatment significantly reduced the DNA damage induced either by glucolipotoxicity or tunicamycin (Figure 5(a)). Since CHOP (an intermediate in caspase-dependent apoptosis) mRNA and protein expression were increased in cells subjected to glucolipotoxicity or tunicamycin, we estimated the activity of caspase-3 enzyme. As expected, cells treated with glucolipotoxicity or tunicamycin showed significantly increased caspase-3 activity (Figure 5(b)) implying that uncontrolled ER stress might have programmed the cells towards apoptosis. Increased caspase-3 activity either due to glucolipotoxicity or tunicamycin was significantly (*P* < 0.05) reduced by 4-PBA.

## 4. Discussion

The following are the nutshell findings of the study. First, monocytes subjected to glucolipotoxicity showed increased ROS generation and increased mRNA expression of several genes of the ER stress machinery. Secondly, these monocytes exhibited features of chronic ER stress as evidenced by increased mRNA expression of TRPC-6, DNA damage, and caspase-3 activity. Thirdly, PBA—a chemical chaperone, resisted all the glucolipotoxicity-induced cellular and molecular alterations in monocytes emphasizing the benefits of ER stress alleviation.

Monocyte activation, adhesion to the endothelium, and transmigration into the subendothelial space are key events in early pathogenesis of atherosclerosis. The mechanisms by which glucose and lipid toxicity induces monocyte-associated atherosclerosis are only partially known. Mononuclear blood cells from patients with diabetes show increased generation of reactive oxygen species and altered redox signaling [16–21]. In our study, monocytes subjected to glucolipotoxicity exhibited increased generation of ROS. ROS could activate cell death processes directly by the oxidation of proteins, lipids, and/or nucleic acids or could act as initiators or second messengers in the cell death process. Accumulating evidence suggests that protein folding and generation of reactive oxygen species (ROS) as a byproduct of protein oxidation in the ER are closely linked events. It has also become apparent that activation of the UPR on exposure to oxidative stress is an adaptive mechanism to preserve cell function and survival. However, persistent oxidative stress and protein misfolding initiate apoptotic cascades and are now known to play predominant roles in the pathogenesis of multiple human diseases including diabetes and atherosclerosis [22–24].

A number of hypotheses have been conceived to explain advanced lesional macrophage apoptosis and atherosclerosis, and undoubtedly more than one mechanism is involved. Recent mechanistic data in cultured cells and correlative and genetic causation evidence *in vivo* support a role for endoplasmic reticulum (ER) stress in advanced lesional macrophage apoptosis and its major consequence of plaque necrosis [13, 25]. Consistent with previous findings, glucolipotoxicity in our study showed increased transcription of PERK, GRP78, IRE1*α*, XBP-1, ATF6, and CHOP in monocytes. This was also corroborated by the increased protein expression of ER stress markers. The fact that increased expression of ER stress markers under glucolipotoxicity is similar to that induced by tunicamycin (a known inducer of ER stress) emphasizes that glucolipotoxicity inducts ER stress in monocytes.

Increased TRPC-6 mRNA expression under glucolipotoxicity in our study implies a role for increased calcium levels in monocyte dysfunction and ER stress. Calcium is an essential intracellular messenger and serves critical cellular functions in both excitable and nonexcitable cells. Available data on transient receptor potential conical (TRPC) protein indicate that these proteins initiate Ca^2+^ entry pathways and are essential in maintaining cytosolic, ER, and mitochondrial Ca^2+^ levels [26]. Alterations in Ca^2+^ homeostasis have been suggested in diabetes and associated complications [27, 28]. Zhu et al. [28] have recently reviewed a role for TRP channels and their implications in metabolic diseases. Activity of TRPC is physiologically important as Ca^2+^ concentrations within the ER must be maintained at sufficient levels in order for the organelle to carry out many of its fundamental functions including protein folding and trafficking. However, loss of Ca^2+^ homeostasis due to improper TRPC activation could lead to ER stress responses, and even apoptosis [29]. While oxidative stress could result in cellular defects including a defect in ER Ca^2+^ uptake and Ca^2+^ efflux, thereby increasing [Ca^2+^]_i_ and Ca^2+^ influx [30], several TRPC families have also been shown directly activated in response to oxidative stress [31]. In our study, both glucolipotoxicity and tunicamycin resulted in increased ROS generation in monocytes and, at the transcription level, we have also witnessed the increased TRPC6 mRNA expression. Among the TRPC subfamily of TRP channels, TRPC6 is gated by signal transduction pathways that activate C-type phospholipases as well as by direct exposure to diacylglycerols [32]. Recent studies emphasize a pathophysiological role of TRPC6 in several disease states [33, 34] and it appears to be an emerging drug target. Altered TRPC6 regulation and impaired capacitative calcium entry have been shown in vessels of diabetic patients [35]. While a role for oxidative stress has been implicated in the pathogenesis of Type 2 diabetes and its micro- and macrovascular complications [20], TRPC6 mRNA was also shown significantly higher in monocytes from patients with type 2 diabetes [36] pointing to a novel pathway for increased activation of monocytes and hence atherosclerosis in patients with diabetes.

ER stress is one of the damaging stresses resulting from glucolipotoxicity which can lead to apoptosis when the stress is prolonged or uncontrolled. Komura et al. [37] have shown that monocytes of diabetic patients are not as efficient in phagocytosing as in normal healthy people and electron microscopic examination of monocytes revealed morphologic alterations in the ER of cells derived from patients with diabetes. Although it has been known that activation of the CHOP pathway of the UPR can cause apoptosis, the molecular mechanisms linking CHOP to death execution pathways is poorly understood. Our results here show that apoptosis might be executed by the increased activation of caspase-3 under glucolipotoxicity. Since autophagy has been proposed to operate as an alternative cell death mechanism or act upstream of apoptosis [38], it is plausible that glucolipotoxicity-induced ER stress could also promote cell death via autophagy [39, 40]. ER stress-induced UPR activation can also influence the expression of certain inflammatory cytokines [41] rendering the monocyte intrinsically proinflammatory which might have drastic consequences when monocytes infiltrate the intima of the vessel wall. Recent work has provided evidence for a calcium dependent mechanism in ER stress-induced macrophage apoptosis [42]. The increased mRNA levels of TRPC-6 seen in our study also support this. Since caspase and the downstream apoptosis effector molecules are Ca^2+^-dependent, future studies should also focus on the involvement of intracellular calcium in the induction of apoptosis in ER-stressed cells under glucose and lipid dyshomeostasis.

In our study, glucolipotoxicity-induced ROS generation and increased ER stress markers seen under glucolipotoxicity were normalized by PBA. Drugs that interfere with ER stress have wide therapeutic potential and recently chemical chaperones like tauroursodeoxycholic acid (TUDCA), and PBA received much attention because of their ER stress alleviating activities as these compounds improve ER folding capacity and help in stabilizing protein conformation [43]. PBA was found to be protective in *in vitro* and *in vivo* models of diabetes [43–45]. Accumulating evidence suggests that protein folding and generation of reactive oxygen species (ROS) as a byproduct of protein oxidation in the ER are closely linked events. While facilitating appropriate protein folding, PBA helps maintaining the balance between ER oxidoreductin 1 (ERO1) and protein disulfide isomerase (PDI) and thereby reduces the ROS levels generated during protein oxidation [46]. It has also been shown that PBA counteracts oxidative stress by upregulating the expression and activity of superoxide dismutase (SOD) [47, 48]. Luo et al. [49] have also demonstrated PBA inhibition of NADPH oxidase activity and emphasized a dual regulation ER stress and oxidative stress by PBA. Erbay et al. [50] demonstrated that mitigation of ER stress with PBA protected macrophages against lipotoxic death and atherosclerosis by suppression of XBP1 splicing and CHOP expression. PBA has been shown to regulate ER stress and offer potential therapeutic benefits in several preclinical models of human diseases including type 2 diabetes [43, 51–55]. As an orally bioavailable terminal aromatic substituted fatty acid, PBA has been used for the treatment of urea cycle disorders [56]. More importantly, oral treatment of PBA was recently shown preventing lipid-induced impairment in insulin sensitivity and b-cell function in humans [57].

To conclude, our study exposes the convergence of ER stress, oxidative stress and apoptosis in the presence of glucolipotoxicity in monocytes and points out ER stress network as a novel drug-targetable pathway. Our results also emphasize that chemical chaperones enhance the adaptive capacity of the ER, and on further evaluation in appropriate clinical trials, could serve as potent antidiabetic/antiatherosclerotic modalities with potential application in the treatment of type 2 diabetes and its vascular complications like atherosclerosis.

## Figures and Tables

**Table 1:** Primer sequence of specific genes.

GRP78	Forward CTG CCA TGG TTC TCA CTA AAA TG
	Reverse TTA GGC CAG CAA TAG TTC CAG

PERK	Forward GAA CCA GAC GAT GAG ACA GAG
	Reverse GGA TGA CAC CAA GGA ACC G

IRE1	Forward GCG AAC AGA ATA CAC CAT CAC
	Reverse ACC AGC CCA TCA CCA TTG

XBP1	Forward TGG ATT CTG GCG GTA TTG AC
	Reverse TCC TTC TGG GTA GAC CTCTG

ATF-6	Forward CCT GTC CTA CAA AGT ACC ATG AG
	Reverse CCT TTA ATC TCG CCT CTA ACC C

CHOP/GADD	Forward GTA CCT ATG TTT CAC CTC CTG G
	Reverse TGG AAT CTG GAG AGT GAG GG

TRPC6	Forward TTT GAG GAG GGC AGA ACA CTT CCT
	Reverse TAT GGC CCT GGA ACA GCT CAG AAA

*β*-actin	Forward GTC TTC CCC TCC ATC GT
	Reverse CGT CGC CCA CAT AGG AAT

## Abbreviations

GRP-78: Glucose regulated protein-78
PERK: PKR like ER kinase
IRE-1*α*: Inositol Requiring enzyme-1*α*
XBP-1: X box binding Protein
ATF-6: Activating transcription factor-6
CHOP: CCAAT/enhancer-binding Homologous protein
GADD: Growth arrest and DNA damage protein
TRPC-6: Transient receptor protein channel-6.
